# Prevalence and Factors Associated with Perceived Stigma among Patients with Epilepsy in Ethiopia

**DOI:** 10.1155/2015/627345

**Published:** 2015-09-06

**Authors:** Tolesa Fanta, Telake Azale, Dawit Assefa, Mekbit Getachew

**Affiliations:** ^1^Amanuel Mental Specialized Hospital, Addis Ababa, Ethiopia; ^2^Department of Psychiatry, Faculty of Medicine and Health Science, University of Gondar, Gondar, Ethiopia

## Abstract

*Background*. Epilepsy stigma is considered to be one of the most important factors that have a negative influence on people with epilepsy. Among all types of stigma perceived stigma further exerts stress and restricts normal participation in society. *Methods*. Hospital based cross-sectional study was conducted from May 1, 2013, to May 30, 2013. All patients with epilepsy in Ethiopia were source population. The sample size was determined using single population proportion formula and 347 subjects were selected by using systematic random sampling method. Data was analyzed by using SPSS version 20. *Results*. A total of 346 participants with mean age of 29.3 ± 8.5 SD participated with a response rate of 99.7%. The prevalence of perceived stigma was 31.2%. Age range between 18 and 24 [AOR = 2.84, 95%CI: 1.02, 7.92], difficulty to attend follow-up because of stigma [AOR = 3.15, 95%CI: 1.19, 8.34], seizure related injury [AOR = 1.88, 95%CI: 1.12, 3.15], and contagion belief [AOR = 1.88, 95%CI: 1.10, 5.08] were significantly associated with perceived stigma. *Conclusions*. Perceived stigma was found to be a common problem among patients suffering from epilepsy. The results reinforce the need for creating awareness among patients with epilepsy and addressing misconceptions attached to epilepsy.

## 1. Introduction

Epilepsy is a chronic brain disorder characterized by recurrent derangement of the nervous system due to sudden excessive discharge of the cerebral neurons [1]. It is a neurological condition that knows no geographic, social, or racial boundaries, occurring in men and women and affecting people of all ages [2]. It affects approximately 50 million people worldwide [3].

The population prevalence of epilepsy varies across countries from 0.5 to 5%. The higher levels tend to be seen in developing countries where fewer than 50% of cases are on medication [4]. In a large community-based epidemiological study, the prevalence of epilepsy in Ethiopia was reported as 5.2/1000 population [5, 6]. The incidence was 64/100,000 population as reported in a community-based study conducted in Mescal and Marko districts of rural central Ethiopia [7].

Stigma is social process or related personal experience characterized by exclusion, rejection, blame, and devaluation and is a phenomenon associated with many chronic health conditions. Stigma and its psychosocial consequences cause indescribable suffering to those who are stigmatized. In addition, stigma has indirect but strongly negative implications for public health efforts to combat the diseases concerned. Epilepsy has a considerable psychological and emotional impact on PWE. Uncontrolled seizures can be very unsettling. People may fear even going outside their homes unaccompanied. They may fear what people might think of them if they were to have a seizure in public [8].

Across the world and throughout history, epilepsy has been a culturally devalued condition and awareness about epilepsy is usually very low. Such devaluing and lack of awareness often make people with epilepsy be stigmatized, discriminated, and excluded from society to problems at work and economic difficulties, bearing a psychosocial burden as well as inappropriate treatment [9–21]. From the patient's view, the diagnosis of epilepsy triggers a change in perception, bringing on fears of being different and anxiety about the future in the community, with apprehension about getting a job or starting a family [22–31]. Understanding and reducing epilepsy-associated stigma are one of the stated aims of the World Health Organization's Global Campaign against Epilepsy “Out of the Shadows” initiative [32].

According to study conducted among more than 5,000 patients living in 15 countries in Europe 51% reported feeling stigmatized with 18% reporting feeling highly stigmatized and people with epilepsy who experienced injury during seizure attack reported high feeling of stigma [30]. Another study conducted in Via Christi Comprehensive Epilepsy Center, Wichita, KS, USA, on PWE indicates that about 34% of the respondents who were on AEDs indicated they had belief that the general public had negative feelings and reactions towards individuals with epilepsy [33].

The case-control study conducted in Cambodia reveals that about 46% cases reported highest stigma score [34]. Another institutional based cross-sectional study conducted at Brooklyn Hospital Academic Medical Center on patients with epilepsy reveals that 69% of respondents fulfill the criteria of perceived stigma [35]. According to community-based cross-sectional study conducted in Campinas, Brazil, on 1850 PWE, the median of the general stigma score was 42 and low educational level, female sex, and religion were sociodemographic factors that made great association with perceived stigma [36]. According to the institutional based cross-sectional study conducted in Iran, the Gulf, and Near East on 3,889 people with epilepsy attending epilepsy clinic, about one-third (~33%) of the patients felt stigmatized by their epilepsy [37]. A community-based cross-sectional study conducted in Benin reveals that the proportion of PWE who felt stigmatized by their condition was 68.7%, and 38.8% of them reported high feelings of stigma [38]. According to case-control study conducted in Zambia, Lusaka, on 169 adults who were on follow-up at epilepsy clinic the median stigma score was 2.5 and higher stigma scores were associated with community disclosure, forced disclosure either through a public seizure or through someone else revealing their condition to the community. People who believed epilepsy to be contagious also had higher felt stigma [39]. A descriptive cross-sectional survey conducted in Kilifi, Kenya, on 673 respondents reported that 33% felt stigmatized as measured by the KSSE; and younger age was significantly associated with perceived stigma [40]. According to the institutional based cross-sectional study which was conducted in Butajira, Ethiopia, on 831 respondents the prevalence estimate of perceived stigma was 81%. Male sex and age group of 45 years and above were sociodemographic factors which were significantly associated with PS.

Those who had more frequent seizures also reported experiencing more stigma compared to those who had less frequent seizures [41].

## 2. Objective

The aim of the study is to assess the prevalence of perceived stigma and factors associated with it among patients with epilepsy in Ethiopia.

## 3. Method

Institutional based quantitative cross-sectional study was conducted from May 1, 2013, to May 30, 2013, at Amanuel Mental Specialized Hospital which is the only mental hospital in the country. The minimum number of samples required for this study was determined by using single population proportion formula and found to be 347. The adults aged 18 years and above were included in the study and subjects who had major cognitive impairments or intellectual disability were not included in the study. A systematic random sampling technique was used to select participants. People with epilepsy who were already diagnosed by neuropsychiatrist as having epilepsy and were on regular follow-up at Amanuel Hospital Epilepsy Department were interviewed using the Kilifi Stigma Scale of Epilepsy which was developed and validated in Kilifi, Kenya, with high internal consistency, Cronbach's *α* of 0.91, and excellent test-retest reliability with *r* of 0.92. It is a simple three-point Likert scoring system scored as “not at all” (score of 0), “sometimes” (score of 1), and “always” (score of 2). It has fifteen items and a total score was calculated by addition of all item scores. The score above the value on 66th percentile of the data indicated presence of perceived/felt stigma [40, 42]. Questions used to assess sociodemographic data and other relevant information were designed by the investigator.

There were also some questions to assess clinical factors, patients' belief about a nature of their illness, and disclosure status of the patient. Data was collected by four trained psychiatry nurses and data collectors were supervised by two B.S. nurses. The questionnaire was translated to local language (Amharic) to be understood by all participants and translated back to English. The translation and back translation were done by neuropsychiatrists and neurologists working in Amanuel Hospital. Data was entered, cleaned, and stored by using EPI info version 3.5.1 and then exported into Statistical Package for the Social Sciences (SPSS) version 20 for analysis. Frequency and percentage were used to describe the data. Crude and adjusted OR was analyzed using logistic regression and the level of significance of association was determined at *p* value < 0.05. Ethical clearance was obtained from the Institutional Review Board of College of Medicine and Health Sciences, University of Gondar, and from Amanuel Mental Specialized Hospital. Written informed consent for participation in the study was obtained from participants just after start of the interview.

## 4. Results

A total of 347 patients with epilepsy were recruited in the study. Out of 347 patients with epilepsy 346 responded to the questionnaire with overall response rate of 99.7%.

### 4.1. Sociodemographic Characteristics

All of the respondents were in the age group of 18–57 years with the mean age of 29.3 ± 8.5 SD. Among all participants, 216 (62.4%) were male. The participants from urban area were (268) (77.5%) and the rest were from rural area. Among the total study subjects 232 (67.1%) were Orthodox religion followers and 177 (51.2%) were single (Table 1).

### 4.2. Description of Respondents by Clinical Factors

Majority of the study subjects (150 (43.4%)) were living with epilepsy for more than 11 years and 114 (33%) were on AEDs for more than 11 years. Among the study subjects who had seizure in past three months (81 (23.4%)) majority of them (58 (71.6%)) reported that they experienced seizure less than six times in past three months (Table 2).

### 4.3. Description of Respondents by Disclosure Status, Patient's Contagion Belief, and Causal Belief of Epilepsy

Majority of the respondents (241 (69.7%)) reported that their condition is disclosed to the society, and among these 196 (81.3%) reported that their condition is disclosed involuntarily (attack occurred at public place and/or other persons disclosed their condition). Forty-two percent of the participants believe that the cause of their epilepsy is evil spirit (Table 3).

### 4.4. Perceived Stigma Scores of Participants

**Figure 1:**
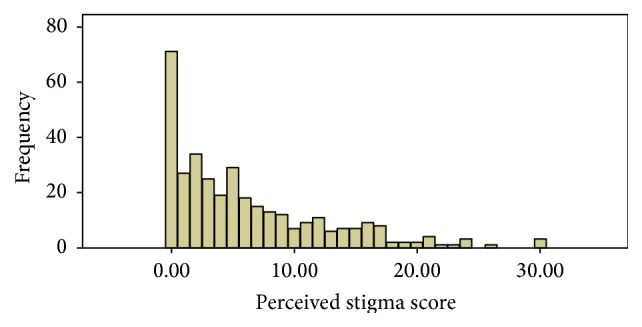
Scores for the Kilifi Stigma Scale for Epilepsy.

Most of the participants reported that they feel disappointed in their self which is followed by feeing embarrassed (Table 4). The lowest score of the data was 0 and the highest score was 30. The lower, median, and upper quartile values were 1, 4, and 9, respectively. The 66th percentile of the data was 7, so that scores above 7 were considered to show the PWE who felt stigmatized (Figure 1). Accordingly, out of the 346 study subjects recruited in the study, 31.2% fulfilled the criteria for perceived stigma as measured by the KSSE.

### 4.5. Multivariate Analysis of Perceived Stigma and Explanatory Variables

The multivariate logistic regression which controls the effect of confounding variables was used by taking all covariates into account simultaneously for perceived stigma. Analysis was done after adjusting for age, residence, educational status, difficulty in attending follow-up, difficulty in taking medication daily, seizure related injury, and contagion belief of epilepsy. Accordingly age group between 18 and 24 years were about 2.8 times more likely to have perceived stigma as compared to age group ≥ 45 years (AOR = 2.84, 95% CI: 1.02, 7.92). Those who reported difficulty in attending follow-up because of stigma were 3 times more likely to have perceived stigma compared to those who have no difficulty in attending follow-up because of stigma (AOR = 3.15, 95% CI: 1.19, 8.34). The patients with epilepsy who had seizure related injury were about 1.8 times more likely to have perceived stigma when compared to those who have no seizure related injury (AOR = 1.88, 95% CI: 1.12, 3.15). The participants who believe that epilepsy is contagious were 2.4 times more likely to have perceived stigma as compared to those who believe that epilepsy is not contagious (AOR = 2.37, 95% CI: 1.10, 5.08) (Table 5).

## 5. Discussion

The study found that the prevalence of perceived stigma among patients with epilepsy in Ethiopia in year 2013 was 31.2%. The prevalence of PS in current study is lower when compared with other studies conducted in Butajira, Ethiopia, which was 81% [41]. The discrepancy might be due to difference in study setting and difference in instrument used. The prevalence of PS in our study is in line with that of Kenya which was 33% [40].

The prevalence of perceived stigma in our study is lower when compared to that of Benin which was 68.7% [38]. The variation might be due to difference in instrument they used which was Jacoby Stigma Scale and difference in study setting. The study conducted in Brazil and Zambia reported that the median stigma score of patients with epilepsy was 42 and 2.5, respectively [36, 39]. The median score of current study which is 4 is lower when compared with median stigma score in Brazil and is higher when compared to that of Zambia. This could be due to instrument difference which was Stigma Scale of Epilepsy in case of Brazil and Jacoby Stigma Scale in case of Zambia. The other reason might be population difference.

The prevalence of perceived stigma reported by our study is lower when compared with the studies conducted in European countries, Cambodia, Kansas, and Brooklyn which was 51%, 46%, 34%, and 69%, respectively [30, 33–35]. This is explained by population difference, instrument difference which was Jacoby Stigma Scale in all, large sample size they used, and difference in study setting. In other ways the prevalence of perceived stigma found in our study is in line with that of Iran, the Gulf, and Near East, which was 33% [37].

Age group between 18 and 24 were more likely to have perceived stigma (AOR = 2.84, 95% CI: 1.02, 7.92) when compared to those who were 44 years and above. This result is in line with that of Kenya [40]. This is explained by older people who were less likely to report feeling stigmatized because discriminatory attitudes towards epilepsy may have less importance to them than younger people. The younger people want to fit in with peers.

Participants who reported that they had difficulty in attending follow-up because of fear of stigma were more likely to have perceived stigma (AOR = 3.15, 95% CI: 1.19, 8.34) when compared to those who had no difficulty in attending follow-up because of fear of stigma. This could be due to fear of enacted stigma if the society knows that they were on follow-up for epilepsy case.

Participants who experienced injury during seizure attack were more likely to feel stigmatized (AOR = 1.88, 95% CI: 1.12, 3.15) compared to those who did not experience injury during seizure attack. This finding is in line with that of European countries [30]. This may be explained by the physical deformity and scars due to the fact that injury may easily disclose their condition. The scar and the deformity may also cause another stigma or potentiate the existing stigma.

The participants who believe that epilepsy is contagious were more likely to have perceived stigma compared to those who believe that epilepsy is not contagious (AOR = 2.37, 95% CI: 1.10, 5.08). This result is in line with that of Zambia [39]. The possible explanation is because they thought that the society also believes that epilepsy is contagious, so they felt that they were stigmatized by society.

Among the variables entered into multivariate analysis, residence, educational status, difficulty in taking AEDs daily because of fear of stigma, and disclosure status were not associated with perceived stigma.

## 6. Conclusion

The prevalence of perceived stigma, even if it seems low, is not negligible and is showing a significant public health issue among patients with epilepsy that requires great emphasis. Age, difficulty in attending follow-up, seizure related injury, and contagion belief were significantly associated with perceived stigma among patients with epilepsy.

## Figures and Tables

**Table 1:** Distribution of study subjects by sociodemographic factors.

Variables	(*n*/346)	(%)
Age		
18–24	112	32.4
25–34	136	39.3
35–44	57	16.5
≥45	41	11.8
Sex		
Male	216	62.4
Female	130	37.6
Residence		
Rural	78	22.5
Urban	268	77.5
Ethnicity		
Amhara	122	35.5
Oromo	110	31.8
Tigre	26	7.5
Gurage	78	22.5
Others	10	2.9
Marital status		
Married	125	36.1
Single	177	51.2
Divorced	23	6.6
Widowed	21	6.1
Religion		
Orthodox	232	67.1
Protestant	52	15
Muslim	57	16.5
Educational status		
No formal education	45	13
1–8	145	41.9
9–12	123	35.5
12+	33	9.5
Occupational status		
Employed	134	38.7
Farmer	50	14.5
Student	49	14.2
Daily laborer	97	28
Others	16	4.6

**Table 2:** Distribution of study subjects by clinical factors.

Variables	(*n*/346)	(%)
Duration of the illness		
≤1 yr	21	6.1
2–5 yr	90	26
6–10 yr	85	24.6
≥11 yr	150	43.4
Duration on AEDs		
≤1 yr	56	16.2
2–5 yr	106	30.6
6–10 yr	70	20.2
≥11 yr	114	32.9
Seizure in last 3 m		
No	265	76.6
Yes	81	23.4
Frequency of seizure in three months		
≤6x/3 m	58	71.6
>6x/3 m	23	28.4
Difficulty in attending follow-up because of fear of stigma		
No	312	90.2
Yes	34	9.8
Difficulty in taking AEDs daily because of fear of stigma		
Yes	28	8.1
No	318	91.9
Injury during seizure		
No	212	61.3
Yes	134	38.7

**Table 3:** Distribution of study subjects by disclosure status, patient's contagion belief, and causal belief of epilepsy.

Variables	(*n*/346)	(%)
Cause of epilepsy		
Supernatural force	90	26
Evil spirit	147	42.5
Other	15	4.3
I do not know	94	27.2
Contagion belief		
No	306	88.4
Yes	40	11.6
Disclosure status		
Not disclosed	105	30.3
Disclosed	241	69.7
Way of disclosure		
Voluntary disclosure	45	18.7
Forced disclosure	196	81.3

**Table 4:** Proportion of responses to KSSE by study participants.

	Items	*n*/346	%
1	Do you feel different from other people?	119	34.4

2	Do you feel lonely?	125	36.1

3	Do you feel embarrassed?	166	48

4	Do you feel disappointed in yourself?	181	52.3

5	Do you feel that you cannot have a rewarding life?	135	39

6	Do you feel that you cannot contribute anything in society?	86	24.8

7	Do you feel that you cannot join others in public places?	106	30.6

8	Do you feel that other people are uncomfortable with you?	105	30.4

9	Do you feel that other people do not want to go to occasions with you?	80	23.1

10	Do you feel that other people treat you like an inferior person?	110	31.8

11	Do you feel that other people would prefer to avoid you?	106	30.6

12	Do you feel that other people avoid exchanging greetings with you?	62	17.9

13	Do you feel that you are mistreated by other people?	60	17.3

14	Do you feel that other people discriminate against you?	83	24

15	Do you feel that other people treat you like an outcast?	51	14.7

**Table 5:** Factors associated with perceived stigma of people with epilepsy (bivariate and multivariate analysis).

Explanatory variables	Perceived stigma		Bivariate and multivariate analysis	
	Yes	No	COR (95% CI)	AOR (95% CI)
Age				
18–24	38	74	2.49 (1.01, 6.15)	**2.84** (**1.02**, **7.92**)^**∗∗**^
25–34	40	96	2.02 (0.83, 4.94)	2.23 (0.82, 6.06)
35–44	23	34	3.29 (1.25, 8.67)	3.04 (0.34, 9.13)
≥45	7	34	1.00	1.00
Educational status				
No formal education	22	23	2.99 (1.11, 8.03)	2.52 (0.84, 7.58)
1–8	49	96	1.59 (0.67, 3.79)	1.24 (0.47, 3.28)
9–12	29	94	0.96 (0.39, 2.37)	0.72 (0.27, 1.96)
12+	8	25	1.00	1.00
Residence				
Rural	33	45	1.89 (1.12, 3.18)	0.65 (0.34, 1.22)
Urban	75	193	1.00	1.00
Difficulty in attending follow-up				
Yes	23	11	5.58 (2.61, 11.95)	**3.15** (**1.19**, **8.34**)^**∗**^
No	85	227	1.00	1.00
Difficulty in taking medication daily				
Yes	18	10	4.56 (2.03, 10.26)	2.02 (0.70, 5.81)
No	90	228	1.00	1.00
Injury during seizure attack				
Yes	53	81	1.87 (1.18, 2.93)	**1.88** (**1.12**, **3.15**)^**∗**^
No	55	157	1.00	1.00
Contagion belief				
Yes	20	20	2.48 (1.27, 4.83)	**2.37** (**1.10**, **5.08**)^**∗**^
No	88	218	1.00	1.00

^*∗*^Statistically significant at *p* value <0.05. ^*∗∗*^Statistically significant at *p* value <0.001.

## Abbreviations

AEDs: Antiepileptic drugs
AMSH: Amanuel Mental Specialized Hospital
KSSE: Kilifi Stigma Scale of Epilepsy
PS: Perceived stigma
PWE: People with epilepsy.
